# Characterization and adaptation of *Caldicellulosiruptor* strains to higher sugar concentrations, targeting enhanced hydrogen production from lignocellulosic hydrolysates

**DOI:** 10.1186/s13068-021-02058-x

**Published:** 2021-10-30

**Authors:** Eoin Byrne, Johanna Björkmalm, James P. Bostick, Krishnan Sreenivas, Karin Willquist, Ed W. J. van Niel

**Affiliations:** 1 Division of Applied Microbiology, Lund University, PO Box 124, 221 00 Lund, Sweden; 2 RISE, Ideon Science Park, Building Beta 2 3v Scheelevägen 17, 22370 Lund, Sweden; 3 Present Address: Department of Food Biosciences, Teagasc Food Research Centre, Moorepark, Fermoy, Co. Cork P61 C996 Ireland; 4 Present Address: Coriolis Pharma Research GmbH, Fraunhoferstrasse 18B, 82152 Planegg, Germany

**Keywords:** Osmolarity, *Caldicellulosiruptor*, Biohydrogen, Kinetic model, Adaptive laboratory evolution

## Abstract

**Background:**

The members of the genus *Caldicellulosiruptor* have the potential for future integration into a biorefinery system due to their capacity to generate hydrogen close to the theoretical limit of 4 mol H_2_/mol hexose, use a wide range of sugars and can grow on numerous lignocellulose hydrolysates. However, members of this genus are unable to survive in high sugar concentrations, limiting their ability to grow on more concentrated hydrolysates, thus impeding their industrial applicability. In this study five members of this genus, *C.* *owensensis*, *C. kronotskyensis*, *C.* *bescii*, *C.* *acetigenus* and *C.* *kristjanssonii*, were developed to tolerate higher sugar concentrations through an adaptive laboratory evolution (ALE) process. The developed mixed population *C.* *owensensis* CO80 was further studied and accompanied by the development of a kinetic model based on Monod kinetics to quantitatively compare it with the parental strain.

**Results:**

Mixed populations of *Caldicellulosiruptor* tolerant to higher glucose concentrations were obtained with *C.* *owensensis* adapted to grow up to 80 g/L glucose; other strains in particular C. *kristjanssonii* demonstrated a greater restriction to adaptation. The *C.* *owensensis* CO80 mixed population was further studied and demonstrated the ability to grow in glucose concentrations up to 80 g/L glucose, but with reduced volumetric hydrogen productivities ($Q_{{{\text{H}}_{2} }}$) and incomplete sugar conversion at elevated glucose concentrations. In addition, the carbon yield decreased with elevated concentrations of glucose. The ability of the mixed population *C.* *owensensis* CO80 to grow in high glucose concentrations was further described with a kinetic growth model, which revealed that the critical sugar concentration of the cells increased fourfold when cultivated at higher concentrations. When co-cultured with the adapted *C.* *saccharolyticus* G5 mixed culture at a hydraulic retention time (HRT) of 20 h, *C.* *owensensis* constituted only 0.09–1.58% of the population in suspension.

**Conclusions:**

The adaptation of members of the *Caldicellulosiruptor* genus to higher sugar concentrations established that the ability to develop improved strains via ALE is species dependent, with *C.* *owensensis* adapted to grow on 80 g/L, whereas *C.* *kristjanssonii* could only be adapted to 30 g/L glucose. Although *C.* *owensensis* CO80 was adapted to a higher sugar concentration, this mixed population demonstrated reduced $Q_{{{\text{H}}_{2} }}$ with elevated glucose concentrations. This would indicate that while ALE permits adaptation to elevated sugar concentrations, this approach does not result in improved fermentation performances at these higher sugar concentrations. Moreover, the observation that planktonic mixed culture of CO80 was outcompeted by an adapted *C.* *saccharolyticus*, when co-cultivated in continuous mode, indicates that the robustness of CO80 mixed culture should be improved for industrial application*.*

## Background

The current reliance on fossil fuels as the main source of global energy production is not sustainable. Biofuels derived from renewable sources are an extensively researched alternative for the production of energy. It is of great importance these fuels do not compete with food production in terms of land usage [39]. Within the European Union, current legislation restricts dedicated biofuel production to 7% of total land use [14]. Lignocellulose is an attractive substrate for biofuel production due to its wide availability, with 1–5 billion tonnes yielded annually [8]. Currently, over 40 million tonnes of this material is generated as a by-product of agriculture and forestry [37] As such, lignocellulose is ideally suited as a substrate for biohydrogen production as lignocellulose obtained from waste streams does not affect land usage or food production.

Biologically derived hydrogen (biohydrogen) has the potential to be an alternative energy carrier as it can be produced from renewable sources such as lignocellulose and only generates water vapor as a by-product when used as a fuel [2]. However, obstacles that limited bioproduction production include requirement of light (photofermentation), lower hydrogen yield in mesophilic bacteria and the presence of catabolite repression [19]. One potential candidate for biohydrogen production is *Caldicellulosiruptor* and has been previously utilized to generate hydrogen from a variety of lignocellulosic material [5, 10, 28].

*Caldicellulosiruptor* is a genus of thermophilic hydrogen producing bacteria capable of yielding hydrogen close to the maximum stoichiometric yield of 4 mol H_2_/mol hexose when growing at their optimum temperature of 70–80 °C [33, 38]. The species of this genus share a genetic similarity of 93–95%, but originate from various geothermal springs or lakes all over the globe. Notably, most members of this genus can metabolize a wide range of carbon sources including an array of mono-, oligo- and polysaccharides [38]. Species such as *C.* *saccharolyticus* and *C.* *owensensis* display the capacity to simultaneously consume hexoses and pentoses without catabolite repression. It is therefore beneficial to an industrial process as both the cellulose and hemicellulose fractions of lignocellulose can be consumed together [4, 44, 46].

Although a promising candidate for industrial biohydrogen production, *Caldicellulosiruptor* experiences several key limitations including the ability to grow in high osmotic conditions, including high sugar concentrations [5, 21, 28]. In its natural environment *Caldicellulosiruptor* does not experience a high degree of osmotic stress and has thus adapted to low osmolarities, maximally of 400–425 mMol, with a critical osmolarity of 270 to 290 mMol. This osmo-sensitivity limits the industrial potential of *Caldicellulosiruptor* as it precludes cultivation in concentrated sugar mixtures, such as lignocellulose hydrolysates. Concentrated hydrolysates are essential for environmentally efficient production of thermophilic H_2_ as higher substrate concentrations reduce the requirement for water addition and energy input for heating [5, 16, 22].

However, one way to improve osmotolerance of microorganisms is through targeting genes involved in responses to increased osmotic pressure through metabolic engineering and has become an intensive research approach [23]. Recently, *C.* *bescii* was investigated to identify its response mechanism to higher osmolarities, which then can be targeted by directed engineering [36]. Alternatively, when genetic engineering tools are missing strain improvement can be accomplished through a process known as adaptive laboratory evolution (ALE). In this process, an organism is repeatedly sub-cultivated under defined conditions enabling a controlled adaptation to these conditions and hence a favorable phenotype change can develop [13].

In this paper, we have attempted to overcome limitation by making *Caldicellulosiruptor* more tolerant to increased glucose concentrations. We have applied this successively with *C.* *saccharolyticus* [27] and here we describe the development of several adapted cultures of other *Caldicellulosiruptor* species, i.e., *C*. *owensensis*, *C.* *kronotsyensis*, *C.* *bescii*, *C.* *acetigenus* and *C.* *kristjanssonii* through sequential ALE at incrementally increasing glucose levels. The adapted *C*. *owensensis* (CO80) was cultivated in controlled batch and exposed to a high concentration of glucose, up to 80 g/L (440 mMol). Finally, *C.* *owensensis* CO80 was further analyzed in co-cultures with the adapted *C.* *saccharolyticus* G5 [27] on defined media and lignocellulosic hydrolysate of which the data have been published elsewhere Byrne et al. [5]. To quantify the success of adaptation development, this process was mathematically modeled using a growth kinetic equation based on Monod with a set of inhibition equations.

We applied the powerful tool of mathematical modeling to assess how the key physical and biological phenomena in a process function. Inhibition arising from sensitivity to sugar concentration can be one such phenomenon and is further addressed in this paper. This modeling of quantitative description of substrate inhibition and inhibition due to a high degree of osmotic stress have previously been studied using different types of growth kinetic equations [1, 7, 12, 43]. A non-competitive equation was applied to our dataset to describe growth inhibition due to substrate or soluble end products [7, 43].

The results below demonstrate that adaptive laboratory evolution can be implemented to facilitate the cultivation of *Caldicellulosiruptor* in media containing 80 g/L glucose, but is highly species dependent. The adapted *C.* *owensensis* CO80 culture was further studied, modeled and implemented into a co-culture with lignocellulose hydrolysates as substrates.

## Results

### Strain development

To assess the ability of different cultures of the *Caldicellulosiruptor* genus to adapt to higher sugar concentrations and to select an adapted one for further development, ALE was undertaken on five species of *Caldicellulosiruptor.* The respective increase in viability at higher sugar concentrations was determined during sequential batches, whereby increased sugar concentration was used as a selective pressure.

**Fig. 1:**
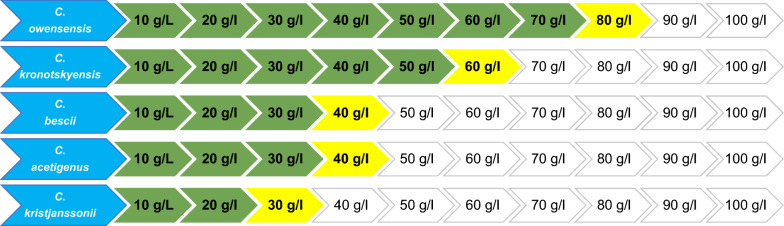
Development of *C. owensensis*, *C. kronotskyensis*, *C. bescii*, *C. acetigenus* and *C. kristjanssonii* adapted to higher sugar concentrations. Values in green indicate adaptation steps were completed in flasks on stated concentrations of glucose. Values in yellow indicate the final adaptation step and therefore the highest concentration of glucose that the species can be grown

The ALE design replicated a previous study that achieved the selection of a *C.* *saccharolyticus* strain with the capacity to grow on 100 g/L glucose [27]. Out of the five selected species, only *C.* *owensensis* was successfully adapted to grow on a glucose concentration of 80 g/L (Fig. 1) over the course of approximately 250 generations. The adaptation of *C.* *kronotskyensis* demonstrated viability in solutions up to 60 g/L glucose but at 70 g/L it did not reach the threshold value of OD_620_ 0.4 and therefore was not selected for further analysis. In contrast, the adaptation strategy of *C.* *kristjanssonii*, *C.* *bescii* and *C.* *acetigenus* was quite restrictive. Even with repeated cultivation at lower sugar concentrations a loss of viability occurred. *C.* *kristjanssonii* was particularly sensitive to adaptation and exhibited poor viability in glucose concentrations as low as 20 g/L. Overall, *C.* *owensensis* had a greater ability to adapt to higher sugar concentrations than any other strain. Adaptation of *C.* *owensensis* to 100 g/L glucose was attempted, however, strains adapted to 90 and 100 g/L displayed poor growth and a loss of viability after several rounds of cultivation. Therefore, the *C.* *owensensis* culture adapted to 80 g/L glucose (CO80) was selected for further analysis.

Adaptation to higher sugar concentrations must be compensated for intracellularly. In general, adaptation of bacterial cells to higher osmolarities is related to intracellular accumulation of compatible solutes and therefore a focused bioinformatics study was performed [18]. However, similar to *C.* *saccharolyticus* [45], *C.* *owensensis* lacks key metabolic pathways for the synthesis of compatible solutes for high osmotic conditions. *C.* *owensensis* lacks synthetic pathways for the osmoprotectants glycine betaine, ectoine and trehalose. *C.* *owensensis* also lacks pathways associated with the synthesis of compatible solutes in thermophiles such as the di‐myo‐inositol phosphate pathway [17, 24] and the synthesis pathway for 2-*O*-(β)-mannosylglycerate as found in *Thermus* *thermophilus* [25]. In addition, no homology between the *C.* *owensensis* genome and 2-(*O*-β-d-mannosyl)-di-myo-inositol-1,3′-phosphate synthase (TM0359) in *Thermotoga* *maritima* [34] could be found*.* However, *C.* *owensensis* can produce glutamate and has the full synthetic pathway of proline.

### Quantitative analysis of CO80 growth at higher sugar concentrations

**Fig. 2:**
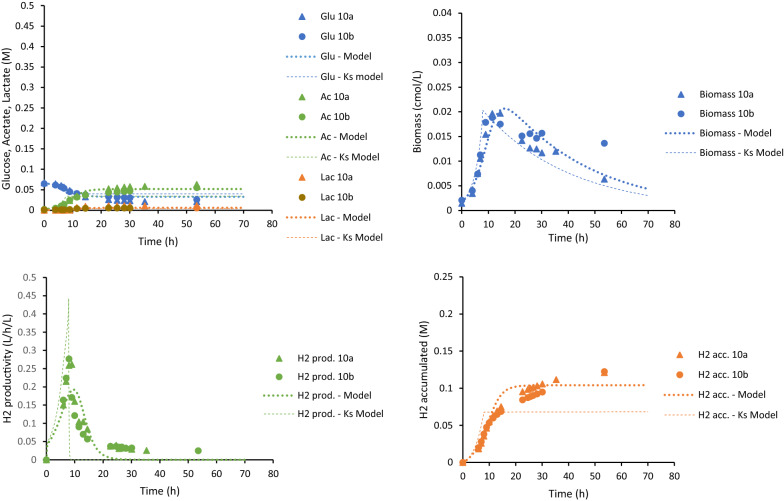
Experimental data (discrete points) and modeling results (lines) for the 10 g/L glucose batch cultures. Upper left: glucose consumption, acetate and lactate production. Upper right: biomass production. Lower left: hydrogen productivity. Lower right: accumulated hydrogen production

**Fig. 3:**
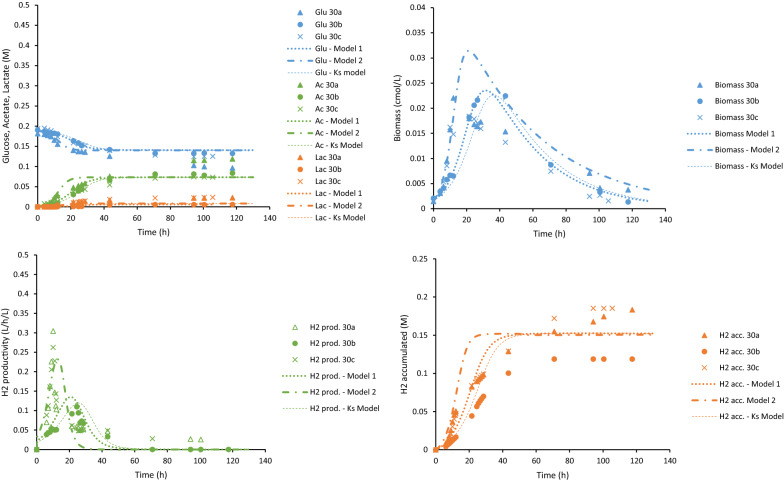
Experimental data (discrete points) and modeling results (lines) for the 30 g/L glucose batch cultures. Upper left: glucose consumption, acetate and lactate production. Upper right: biomass production. Lower left: hydrogen productivity. Lower right: accumulated hydrogen production

**Fig. 4:**
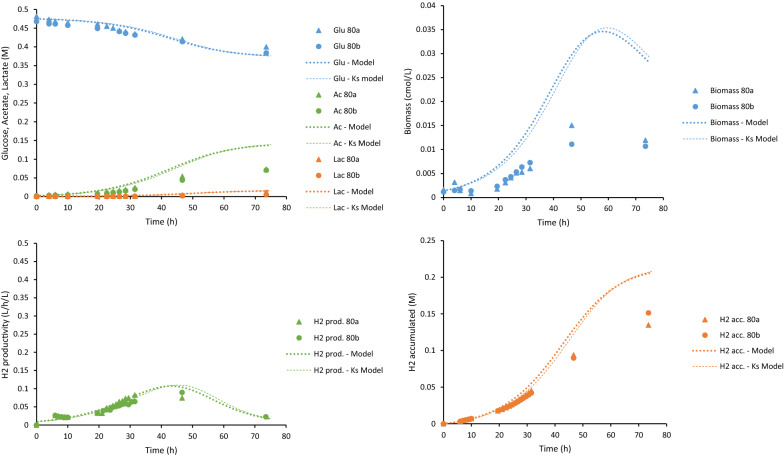
Experimental data (discrete points) and modeling results (lines) for the 80 g/L glucose batch cultures. Upper left: glucose consumption, acetate and lactate production. Upper right: biomass production. Lower left: hydrogen productivity. Lower right: accumulated hydrogen production

*C.* *owensensis* CO80 culture was successfully cultivated on 10, 30 and 80 g/L using a controlled batch reactor (Table 1). The trends of sugar consumption, growth and product formation in these cultures on these different sugar concentrations were monitored (Figs. 2, 3 and 4).

**Table 1:** Comparison of product yields and carbon and redox balances of *C.* *owensensis* DSM 13100 and CO80 batch cultivated in various glucose concentrations

	*C.* *owensensis* DSM 13100^a^	*C.* *owensensis* CO80	*C.* *owensensis* CO80	*C.* *owensensis* CO80
Initial glucose concentration	10 g/L	10 g/L	30 g/L	80 g/L
Yield H_2_ (mol/mol glucose)	4.0 ± 0.2	2.90 ± 0.40	2.31 ± 0.35	1.75 ± 0.10
Yield CO_2_ (mol/mol) glucose	2.3 ± 0.2	1.42 ± 0.12	1.24 ± 0.10	0.91 ± 0.05
Yield acetate (mol/mol glucose)	1.2 ± 0.1	1.41 ± 0.19	1.30 ± 0.21	0.88 ± 0.04
Yield lactate (mol/mol glucose)	0.10 ± 0.01	0.03 ± 0.01	0.23 ± 0.13	0.11 ± 0.05
Residual glucose (g/L)	Not reported	4.24 ± 0.63	21.25 ± 3.43	70.61 ± 2.08
Carbon balance (%)	102 ± 4	84.6 ± 2.0	77.7 ± 4.6	51.9 ± 3.6
Redox balance (%)	97 ± 2	85.4 ± 3.5	75.0 ± 4.7	51.4 ± 3.7

^a^Data from Zeidan and van Niel [46]

Although adaptation of *C.* *owensensis* to higher glucose concentrations permits cultivation at higher glucose concentrations, the adapted strain demonstrated a lower yield of H_2_ than the wild-type strain (Table 1). In addition, when CO80 was cultivated in higher concentrations of glucose a lower carbon and redox balance occurred indicating the production of a yet unknown metabolic product, a reduction in cell mass due to the high rate of cell death, a reduction of glucose concentrations due to Maillard reactions or a combination of these factors.

The behavior of the CO80 culture at increasing glucose concentrations was quantified using dynamic simulations. In these simulations, the model and parameters derived from the wild-type strain of *C.* *saccharolyticus* were used as a benchmark [21]. However, using these parameter values made it clear that the model was inadequate to describe the experimental data. Especially OSM_crit_, which include mainly the glucose, acetate and lactate concentrations, and the rate of death (*r*_cd_) were higher than the benchmark values. This indicated that even if the tolerance to sugar concentrations was higher for the CO80 culture, its cell death was more pronounced than for the wild-type *C.* *saccharolyticus*. In addition, the benchmark values for the maximum specific growth rate (*µ*_max_), affinity constant for glucose (*K*_s_) required some fine-tuning to fit the data points. The adjusted model was calibrated with all data from the duplicates or triplicates of the three batch experiments supplemented with 10 g/L, 30 g/L and 80 g/L glucose. The calibrated parameters are summarized in Table 2. Comparison between the model and the experimental results is graphically shown in Figs. 2, 3 and 4.

**Table 2:** Parameters calibrated to experimental data of *C.* *owensensis* CO80 batch cultures in comparison to the benchmark parameter values of *C.* *saccharolyticus* cultures from Ljunggren et al. [21]

Parameter	Benchmark values Ljunggren et al. [21]	10 g/L	30 g/L^a^	30 g/L^b^	80 g/L
*µ*_max_ (h^−1^)	0.28	0.33 ± 0	0.31 ± 0.082	0.31 ± 0.082	0.29 ± 0.02
*K*_S_ (mol/L)	4.8 × 10^–5^	4.8 × 10^–3^	9.8 × 10^–2^ ± 1.5 × 10^–4^	4.8 × 10^–5e^	0.49 ± 0.064
OSM_crit_ (mol/L)	0.28	0.23 ± 0.0002	0.39 ± 0.002	0.39 ± 0.002	0.78 ± 0.024
*r*_cd_ (h^−1^)	0.014	0.031 ± 0.0001	0.031 ± 0.0065	0.020 ± 0.00015	0.031^c^
$Y_{{S,{\text{H}}_{2} }}$ (mol/mol)	4.77	3.5 ± 0.38	3.5 ± 0.12	3.5 ± 0.12	2.56^c^
*Y*_*S*,*X*_ (cmol/mol)	4.78	0.79^d^	0.80^d^	0.80^d^	0.72^c^
$n_{{{\text{H}}_{2} }}$	4.5	5.37 ± 0.00005	5.37^c^	5.37^c^	4.5^e^
*n*_µ_	4.68	4.68^e^	4.68^e^	4.68^e^	4.68^e^

Confidence interval 95% is given for those parameters which have been fitted numerically

^a^First model for the 30 g/L cultures

^b^Second model for the 30 g/L cultures

^c^Graphically calibrated

^d^Calculated from experimental data

^e^Same value as in Ljunggren et al. [21]

The maximum hydrogen productivity from the experimental data was 10.55 ± 0.04, 11.45 ± 0.00 and 3.35 ± 0.00 mmol/L/h for 10, 30 and 80 g/L sugar, respectively. This observation at 10 and 30 g/L is comparable to, but slightly lower than, 15 mmol/L/h described in wild-type *C.* *owensensis* grown on 10 g/L glucose supplemented with 1 g/L yeast extract [46]. The model underestimated the hydrogen productivity slightly in the case of 10 and 30 g/L, but overestimated productivity compared to experimental data of 80 g/L cultures. Similar overestimation was observed with respect to the cell growth on 80 g/L. Nevertheless, the model was able to predict the experimental data adequately.

The accuracy of the model in describing experimental data was assessed (Table 3). The *R*^2^ values describes how well the model could predict the trend over time and the curve slope values of the linear regression (i.e., *k* in *y* = *k*·*x*) are indicating over- or underestimations. For a perfect fit they should both be 1. With respect to most variables, the prediction error was less than 30% indicating good accuracy. The model was also able to accurately predict the trend of the assessed variables with a *R*^2^ value close to 1 in all cases. However, analysis revealed the overestimation of cell growth as well as acetate and lactate production of the cultures on 30 g/L glucose (Table 3).

**Table 3:** *R*^2^ values and curve slope values to describe the fit between average experimental data and simulated data from the models at the same time points

*R*^2^ values/curve slope values (k)	10 g/L		30 g/L			80 g/L	
State variable	Model	*K*_S_ model	Model 1	Model 2	*K*_S_ model	Model	*K*_S_ model
Glu	0.94/0.95	0.67/0.93	0.91/0.96	0.89/0.95	0.83/1.0	0.87/0.99	0.85/0.98
Biomass	0.82/0.96	0.77/1.0	0.50/6.0	0.28/6.8	0.82/4.2	0.96/0.43	0.94/0.43
Acetate	0.97/0.97	0.75/1.2	0.94/1.1	0.96/1.1	0.77/0.85	0.99/0.54	0.99/0.55
Lactate	0.95/1.4	0.60/1.5	0.97/2.0	0.92/2.1	0.83/1.4	0.97/0.54	0.98/0.54
H_2_ accumulated	0.94/0.92	0.51/1.1	0.94/0.88	0.96/0.98	0.76/0.71	0.99/0.72	0.98/0.74
OSM	0.97/0.98	0.57/1.0	0.94/1.0	0.97/1.0	0.75/0.96	0.91/0.40^a^	0.92/0.40^a^

^a^The linear regression does not intersect (0,0)

### Inhibition kinetics

**Fig. 5:**
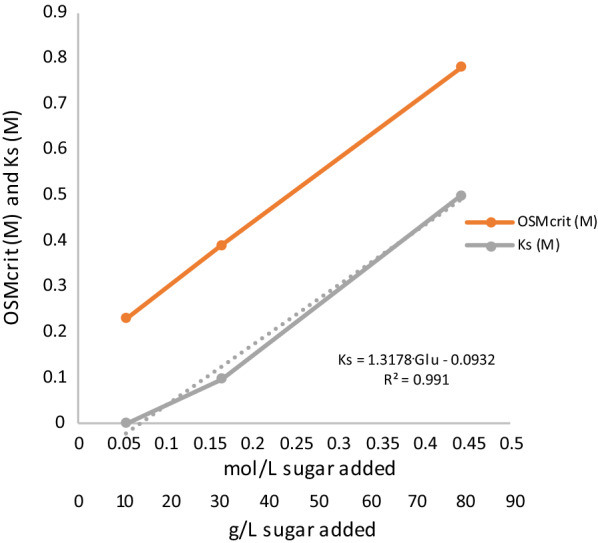
Comparison of the calibrated parameters OSM_crit_ (orange) and *K*_s_ (grey)

The glucose concentration portrayed a linear relationship with the apparent half-saturation constant (*K*_S_) and critical osmolarity (OSM_crit_) (Fig. 5). The apparent *K*_S_ increased with the elevating glucose concentration reaching a value four orders of magnitude higher in the 80 g/L glucose culture. As Sivakumar et al. [40] demonstrated, extraordinarily high *K*_S_ values can be an indicator that the growth kinetics applied is insufficient in describing the process due to substrate inhibition, hence, an extended model was constructed. In the constructed “*K*_S_-model”, the *K*_S_ in the original model (Eq. 7 in “Material and methods”) was replaced with the equation from the linear regression in Fig. 5:

1 $$ \mu = \mu_{\max } \cdot \frac{{{\text{Glu}}}}{{{\text{Glu}} + \left( {1.32 \cdot {\text{Glu}} - 0.09} \right)}} \cdot I_{{{\text{osm}}}} \cdot I_{{{\text{H}}_{{2,{\text{aq}}}} }} , $$

where *µ* is the specific growth rate (h^−1^), *µ*_max_ the maximum specific growth rate (h^−1^), Glu is the glucose concentration (mol/L), *I*_osm_ is the inhibition due to osmolarity and $I_{{{\text{H}}_{{{2},{\text{aq}}}} }}$ is the inhibition due to aqueous hydrogen concentration. The simulation using the “*K*_S_-model” is illustrated in Figs. 2, 3 and 4 as a thin dashed line. The *K*_S_-model was well able to describe the experimental data (Table 3) for 30 g/L and 80 g/L (Figs. 3, 4). However, for 10 g/L, the *K*_S_-model could not sufficiently describe the data (Table 3). This may be due to the greater glucose consumption at 10 g/L compared to the higher concentrations, thereby altering the *K*_S_-model equation to a greater extent than this model is dependent on the glucose concentration.

**Fig. 6:**
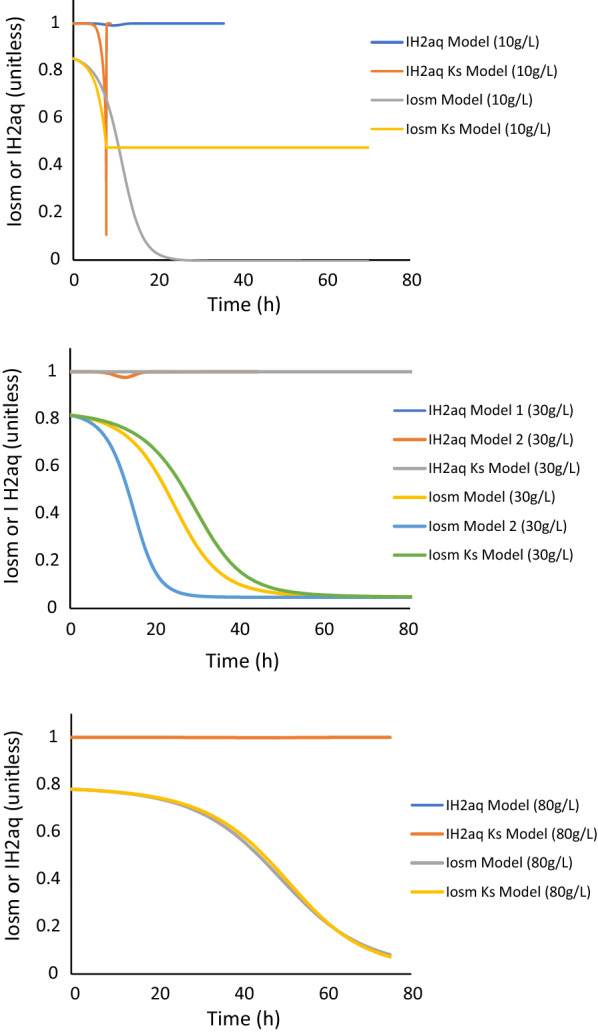
Simulated values of *I*_osm_ and $I_{{{\text{H}}_{{2,{\text{aq}}}} }}$ for the different models

The increase of OSM_crit_ with the sugar concentration (Fig. 5) indicated that the CO80 culture adapted immediately when confronted with a raise in the osmolarity or sugar concentration in the medium. This behavior became more apparent when the inhibition kinetics of the fermentation was simulated in the different cases. The model describes two different types of inhibition, i.e., inhibition by osmolarity (*I*_osm_) and dissolved hydrogen concentration ($I_{{{\text{H}}_{{{2},{\text{aq}}}} }}$) (Eqs. 6 and 7), which were simulated for all three glucose concentrations (Fig. 6). A value around 1 means no inhibition and a lower value means that the process is inhibited. Figure 6 clearly shows that osmolarity is the crucial inhibition factor, i.e., an *I*_osm_ value < 1. $I_{{{\text{H}}_{{{2},{\text{aq}}}} }}$ is of less importance as the simulated values were 0.98 < $I_{{{\text{H}}_{{{2},{\text{aq}}}} }}$ < 1, which means almost no inhibition. Although, the *K*_S_ model for 10 g/L gave values of 0.11 < $I_{{{\text{H}}_{{{2},{\text{aq}}}} }}$ < 1, this rather indicates that the model is not a good fit to the experimental data at this glucose concentration, which confirms what is depicted in Fig. 2. Interestingly, the simulation of *I*_osm_ illustrates that although all fermentations were severely affected by osmolarity, the CO80 culture grown on 80 g/L glucose reached complete inhibition after 80 h, whereas the cultivation on 10 g/L reached complete inhibition after 20 h, although the initial osmolarity in this condition was lower. This indicates that although *C.* *owensensis* CO80 culture is adapted to higher sugar concentrations, it does not manifest the phenotype unless it is stressed in an environment with a high sugar concentration.

It should be noted that at high levels of sugar (80 g/L), significant browning of the media occurred likely due to the presence of Maillard products. This observation could not be quantified and described by the model.

### Reproducibility of the CO80 culture

The model was also used to illustrate the reproducibility of growth of the CO80 culture at increasing sugar concentrations. Three replicates were made for the 30 g/L experiments, as compared to two replicates for the 10 g/L and 80 g/L due to a high degree of variation in one of the replicates. Several attempts at inoculating the CO80 culture to a medium containing 80 g/L glucose failed, as it did not grow when noticeable browning of the media due to Maillard reactions occurred. As illustrated in Fig. 3, one of the three replicates (30b) from the 30 g/L experiments differed with respect to hydrogen productivity and accumulation, but discrepancies could also be seen in the biomass growth. For this reason, a second model (Model 2) with a slight difference in parameter values (Table 2) was constructed for the 30 g/L experiments. However, both Model 1 and Model 2 resulted in low *R*^2^ values and high curve slope values for the biomass (Table 3). One of the three replicates could be simulated with respect to OSM_crit_ and apparent saturation constant (*K*_S_; Fig. 3), whereas the other two could be fitted better with the model where the parameters were much closer to those of the 10 g/L culture. This result might indicate that the adaptation was incomplete, possibly due to the presence of subpopulations possessing different degrees of adaptation to higher sugar concentrations or osmolarity in total [31].

### *Evaluation of CO80 in co-culture*

The results of the batch cultivations indicated that *C.* *owensensis* CO80 was adapted to increased substrate concentrations, but did not grow optimally at these conditions. A further attempt has been made to improve the performance of this strain by co-cultivation with the adapted *C.* *saccharolyticus* G5 culture in defined media and wheat straw hydrolysate, of which the data were published elsewhere [5]. Overall, the co-cultures on wheat straw hydrolysate displayed better performance, such as higher $Q_{{{\text{H}}_{2} }}$ and sugar consumption rates, than on the defined media that contained a sugar composition corresponding to the wheat straw hydrolysate (Table 4).

**Table 4:** Volumetric productivity of continuous co-cultures of *C. owensensis* CO80 and *C. saccharolyticus* G5

	Wheat straw hydrolysate with EB-1	Defined medium with EB-1	Defined medium modified DSM 640
*Q*_glucose_	1.88 ± 0.02	0.18 ± 0.16	0.09 ± 0.13
*Q*_xylose_	2.64 ± 0.39	1.26 ± 0.07	1.49 ± 0.25
*Q*_arabinose_	0.18 ± 0.00	0.20 ± 0.00	0.16 ± 0.00
*Q*_acetate_	4.74 ± 0.00	2.37 ± 0.37	2.63 ± 0.38
$Q_{{{\text{H}}_{2} }}$	6.71 ± 0.06	2.47 ± 0.55	3.71 ± 0.42
Carbon balance	96.8 ± 1.4	101.4 ± 0.9	99.4 ± 5.4
Redox balance	97.8 ± 3.2	100.7 ± 0.0	96.6 ± 5.3

Data adapted from Byrne et al. [5]

The population dynamics of co-cultures were analyzed to determine the stability of the co-cultures. As illustrated in Table 5, only a minute proportion of the co-culture consisted of *C.* *owensensis* CO80 in each case, thus *C.* *saccharolyticus* G5 dominated. However, a brief interruption of pH control during the co-culture on modified DSM 640 resulted in the population of CO80 exceeding 85% of the total population before returning to less than 1% after 2 volume changes. Although low population numbers of planktonic CO80 were observed, a large quantity of biofilm occurred in all continuous cultivations particularly at the gas–liquid interface.

**Table 5:** Population distribution of *C.* *owensensis* C80 and *C.* *saccharolyticus* G5 in continuous cultures

Proportion	Strain G5	Strain C80
Wheat straw hydrolysate	99.76 ± 0.43%	0.24 ± 0.43%
Defined medium EB-1	99.91 ± 0.01%	0.09 ± 0.01%
Defined medium DSM 640	98.45 ± 3.06%	1.58 ± 3.17%

## Discussion

In this study, we successfully implemented ALE as development technique for improving the survival of *C.* *owensensis* at higher sugar concentrations, next to *C.* *saccharolyticus* [27]. *C.* *owensensis* was successfully adapted to survive in 80 g/L glucose. However, not all *Caldicellulosiruptor* strains were as easily adaptable in our study. There were significant restrictions in the adaptation of *C.* *bescii*, *C.* *acetigenus* and *C.* *kristjanssonii* to higher sugar concentrations. *C. bescii* has been previously demonstrated to be completely inhibited by osmolarities above 250 mMol [15]. However, Basen et al. reported that *C. bescii *is capable of growth in media containing 90 g/L (550 mMol), albeit with a lag phase of 50 h [3]. *C.* *kristjanssonii* displayed a particular resistance to adaptation to higher glucose concentrations with a loss of viability above 30 g/L. Previously, a transcriptional analysis demonstrated that adaptation in *C.* *saccharolyticus* was a result of increased transposon activity as well as upregulation of proteins related to sugar transport [27]. However, there is no obvious link between the number of active transposons and the extent of adaptation to higher sugar concentrations. This is illustrated by the fact that the best sugar concentration adapters, *C.* *saccharolyticus* and *C.* *owensensis* have 92 and 32 functional transposons, respectively, which is comparable with those of the worst adapters, *C.* *kristjansonii* and *C.* *bescii* having 57 and 41 functional transposons, respectively. It can be argued whether an adaptation to higher sugar concentration might be related to possessing higher osmotolerance. In a recent study, Sander et al. [36] succeeded in developing two *C.* *bescii* strains possessing higher osmotolerance through genetic engineering. Analyses of their phenotypes resulted in that enhanced tolerance was accomplished through deletion of the FapR, a negative regulator of the fatty acid synthesis. Their analysis further hinted that mutations in regions of the genome of as yet unknown function, also increased osmotolerance, which demands validation. In short, evolvement of higher tolerance to osmotic potential may depend on expression of various (combinations of) genes and may even be species or strain dependent.

Although ALE increased tolerance to higher sugar concentrations, *C.* *owensensis* CO80 exhibited incomplete glucose consumption at elevated concentrations. This phenomenon has been previously observed in wild-type *C.* *saccharolyticus* [27]*.* In addition, when cultivated on 80 g/L glucose, a significantly reduced volumetric hydrogen productivity was obtained compared to 10 and 30 g/L. Additionally, glucose uptake capacity was negatively affected, indicating that although *C.* *owensensis* is capable of surviving at 80 g/L, a significant loss of performance is observed.

The model was shown to be a useful tool to quantify the performance and phenotype of the adapted cultures. In contrast to what was observed with the same model calibrated to data from wild-type *C.* *saccharolyticus* batch cultivations, the sensitivity to osmolarity was the dominating factor over hydrogen inhibition in this condition. A high value of the OSM_crit_ parameter in the model for the CO80 culture indicated a higher tolerance to osmolarity than for the wild-type *C.* *saccharolyticus*. Obviously, during the fermentations it was especially the sugar concentration that dictated the osmolarity. The increase of this parameter was, however, accompanied by a higher death rate in the CO80 phenotype than for the benchmark values and an apparent substrate inhibition kinetics and lower OSM_crit_ at conditions with higher substrates concentrations. In accordance with these results, it is possible that the phenotype of the adapted culture shifted in unison with the osmotic pressure of the environment, implicating the involvement of an active physiological mechanism. Alternatively, since the CO80 culture was obtained through batch-mode cultures, it is in fact not a pure strain but a consortium of strains each adapted to the condition to varying degree. As a result, different strains could have become dominant under the different applied conditions, which led to a difference in the estimations of the parameter values. This could also explain the significantly different growth profiles during repeating batches with 30 g/L. The apparent substrate inhibition kinetics, mainly in cultures at 30 g/L and 80 g/L glucose, may complicate further kinetic analysis of this phenomenon. Due to this inhibition, the apparent *K*_S_ value of the culture with 80 g/L glucose appeared to be four orders of magnitude higher than that of the cells in the culture of 10 g/L glucose (Eq. 7) and in previous studies [7, 21, 43].

The reduction in $Q_{{{\text{H}}_{2} }}$ observed in batch fermentations is consistent with the data derived from Byrne et al. [5] establishing that utilizing adapted cultures facilitated use of more concentrated hydrolysates albeit at the expense of $Q_{{{\text{H}}_{2} }}$. In that study the $Q_{{{\text{H}}_{2} }}$ of the co-culture (6.71 ± 0.06 mmol/L/h) was lower than that observed in pure culture of the wild-type *C.* *saccharolyticus* grown on approximately threefold lower concentrated WSH containing 11 g/L monosaccharides (8.69 mmol/L/h) [28]. However, the $Q_{{{\text{H}}_{2} }}$ obtained with the defined DSM 640 medium was similar to that of wild-type *C.* *saccharolyticus* (4.2 mmol/L/h) [11]. Furthermore, the co-culture grown on WSH displayed a higher $Q_{{{\text{H}}_{2} }}$ when cultivated on wheat straw hydrolysate than on a defined medium. This confirms previous observations that *Caldicellulosiruptor* possesses a higher $Q_{{{\text{H}}_{2} }}$ when cultivated on wheat straw hydrolysate than on pure sugar [28]. This may be due to the presence of additional nutrients and/or oligosaccharides found in the wheat straw compared to that of the defined medium. The reduction of $Q_{{{\text{H}}_{2} }}$ compared to the wild-type *C.* *saccharolyticus* could be due to the presence of higher concentrations of inhibitory compounds that may reduce hydrogen productivity. *C.* *saccharolyticus* is sensitive to HMF and furfural concentrations above 1 and 2 g/L, respectively [10, 26]. Even though higher hydrolysate concentrations were used in the present study, only trace amounts of HMF and furfural were detected. The presence of, yet unknown, compounds in the hydrolysate could have resulted in the inhibition of *Caldicellulosiruptor*. Furthermore, higher concentrations of sugar intensified the occurrence of Maillard reactions, to which *Caldicellulosiruptor* species are very sensitive. A concentration of 80 g/L glucose led to significant browning of the cultivation media and resulted in failure of growth when the coloring arose before inoculation and was presumably also responsible for inconsistencies during cultivation at 30 g/L. Maillard products are known to inhibit the growth of other thermophilic bacterial species such as *Thermotoga* and *Thermoanaerobacter* [10, 42]. Maillard reactions are quite often cited in studies with thermophilic microorganisms and enzymes (e.g., [20, 41]). In addition, our experience and that of others is that xylose more than glucose is prone to be involved in Maillard reactions (e.g., [6]). The presence of Maillard-based products will reduce the efficiency of any large-scale fermentation. One obvious choice for mitigating such reactions would be the omission of cysteine from the cultivation medium or by maintaining a low background sugar concentration in the culture through utilizing fed-batch or continuous cultures as modes of operation.

Additionally, the co-cultivation of *C.* *owensensis* CO80 and *C.* *saccharolyticus* *G5* resulted in a predominantly *C.* *saccharolyticus* *G5* population, with detection of only small quantities of *C.* *owensensis* CO80, although this could indicate cell mass washout of planktonic *C.* *owensensis* CO80. However, a large quantity of biofilm was observed in the bioreactors after termination of each cultivation. Due to that *C.* *owensensis* is known for its ability to form biofilm [32] might point that *C.* *owensensis* CO80 remained significantly present in the fermentations in immobilized form.

## Conclusions

The adaptation of *Caldicellulosiruptor* to higher sugar concentrations through ALE permitted survival at higher sugar concentrations, however, at the cost of $Q_{{{\text{H}}_{2} }}$. Further, even with the ability to withstand higher sugar concentrations, we have shown some phenotype instability and that it is still the overall osmolarity and not the hydrogen inhibition that is the inhibition mechanism that should be addressed in future development of osmotolerant strains. Implementation of co-cultures of *C.* *owensensis* CO80 and *C.* *saccharolyticus* G5 facilitated cultivation of this genus in higher hydrolysate concentrations than previously reported, but even here reduced $Q_{{{\text{H}}_{2} }}$ were observed compared to wild-type *C.* *saccharolyticus* on dilute hydrolysate. It stands to reason that ALE leads to adaptation to the stress parameter to which it is exposed, albeit at the expense of other desired traits. Therefore, a combination of ALE and metabolic engineering as applied in a Design, Build, Test and Learn cycle [35] is a better strategy to construct the desired phenotype of a hydrogen cell factory. The kinetic models developed herein, were able to predict the behavior of growth of the CO80 culture when exposed to 10 and 30 g/L of glucose. The slight overestimation in the models and the growth kinetics of cultures at 80 g/L glucose illustrates that this is the critical boundary beyond which this culture possesses no further capacity for adaptation. The variation in the parameters values at different conditions might pinpoint that CO80 is not a pure culture, but a consortium of adapted strains with a variation in their phenotypes.

In contrast to *C.* *saccharolyticus* [28], *C.* *owensensis* cannot be cultivated without cysteine, as this species lacks the sulfur assimilation pathway [29]. Therefore, co-cultivations of these two species in the absence of cysteine, but with sulfate as the sole sulfur source, could be of interest. In addition, co-cultivation of wild-type strains of *C.* *saccharolyticus* and *C.* *owensensis* could also stimulate biofilm formation [30]. However, this study demonstrated that *C.* *saccharolyticus* G5 completely overtook *C.* *owensensis* CO80 in the co-cultivations. Although this observation can be considered discouraging, large quantities of biofilm occurred indicating the presence of *C.* *owensensis* CO80. Therefore, alternative reactor systems should be applied to enhance biofilm formation, thereby improving cell mass retention that will contribute to higher $Q_{{{\text{H}}_{2} }}$. The co-culture can possible be a strategy to increase the robustness of the bioreactor performance since we observed that CO80 took over at conditions when the bioreactor was acidified. However, for industrial application, the properties of the CO80 mixed population to reach higher hydrogen productivities need to be significantly improved.

## Material and methods

### Strains and cultivation medium

The wild-type strains of *Caldicellulosiruptor* *owensensis* DSM 13100, *Caldicellulosiruptor* *kronotskyensis* DSM 18902, *Caldicellulosiruptor* bescii DSM 6725, *Caldicellulosiruptor* *acetigenus* DSM 7040 and *Caldicellulosiruptor* *kristjanssonii* DSM 12137 were obtained from the Deutsche Sammlung von Mikroorganismen und Zellkulturen (DSMZ; Braunschweig, Germany). Subcultivations were conducted in 250-mL serum flasks with 50 mL modified DSM 640 media [45] with the addition of 50 mM HEPES and 10 g/L glucose, unless otherwise stated. A 1000× vitamin solution was prepared as per Zeidan and van Niel [46] and a modified SL-10 solution was prepared described previously [29].

### Adaptation of species to higher osmolarity

Adaptation of *C*. *owensensis*, *C.* *kronotsyensis*, *C.* *bescii*, *C.* *acetigenus* and *C.* *kristjanssonii* to higher sugar concentrations was performed through adaptive laboratory evolution that initially involved repeated sub-cultivation of each strain in a modified DSM 640 medium containing 10 g/L of glucose. Each flask was incubated to an initial OD of 0.05 and incubated for at least 72 h. Subcultivations were conducted at late-exponential phase and the glucose concentration was increased in 10 g/L increments when generation time for each strain was less than 0.3 h^−1^ and OD was above 0.4. This sequential increase of glucose concentration was continued until no growth in higher glucose concentrations was observed [27].

### Fermentor set-up

Batch cultivations were performed in a jacketed, 3-L fermentor equipped with an ADI 1025 Bio-Console and ADI 1010 Bio-Controller (Applikon, Schiedam, The Netherlands). A working volume of 1 L was used in all batch cultivations and the pH was maintained at 6.5 ± 0.1 by automatic titration with 4 M NaOH. The temperature was thermostatically kept at 70 ± 1 °C. Stirring was maintained at 250 rpm and nitrogen was sparged through the medium at a rate of 6 L/h. A water-cooled condenser was utilized (4 °C) to prevent the evaporation of the medium. During each cultivation, samples were collected at regular intervals for HPLC and to monitor optical density. The supernatant from each sample was collected and stored at − 20 °C for further quantification of sugars, organic acids, and ethanol. Gas samples were collected from the headspace of the fermentor to quantify H_2_ and CO_2_. Analysis of the adapted *C.* *owensensis* CO80 culture was performed using both batch cultivations with the addition of 10, 30 and 80 g/L of glucose. Each of the batch cultivation was conducted in duplicate except for 30 g/L which was performed in triplicate. Co-culturing of *C.* *owensensis* C80 and *C.* *saccharolyticus* G5 in continuous cultures were performed in a previous study [5] at a dilution rate of 0.05 h^−1^. Three different media were used: defined media (modified DSM 640 and EB-1) and wheat straw hydrolysate (for media compositions see Byrne et al. [5]. Biomass samples were taken for population dynamics during steady-state situations.

### Analytical methods

Optical density was determined using an Ultraspec 2100 pro spectrophotometer (Amersham Biosciences) at 620 nm.

Sugars and organic acids were detected using HPLC (Waters, Milford, MA, USA). For the quantification of organic acids, and ethanol, a HPLC was used equipped with an Aminex HPX-87H ion exchange column (Bio-Rad, Hercules, USA) at 60 °C with 5 mM H_2_SO_4_ as mobile phase at a flow rate of 0.6 mL/min. Glucose, xylose and arabinose quantification was conducted using a HPLC with two Shodex SP-0810 Columns (Shodex, Japan) in series with water as a mobile phase at a flow rate of 0.6 mL/min.

H_2_ and CO_2_ concentrations were quantified with an Agilent 7890B Series GC (Agilent GC 7890, Santa Clara, CA) equipped with a TCD detector and a ShinCarbon ST 50/80 UM (2 m × 1/16″ × 1 mm) column. Helium carrier gas was employed, at a flow rate of 10 mL/min. During operation, an initial oven temperature of 80 °C was maintained for 1 min followed by a temperature ramp of 20 °C/min for 4 min with a subsequent 2 min hold time at 160 °C.

### Determination of population dynamics

DNA was extracted from 2 mL of frozen cell pellets—using the GeneJet Genomic DNA purification kit (ThermoFisher, Waltham, MA, USA). qPCR was carried out by amplification of genomic DNA with primers (Table 6) targeting single copy non-homologous regions of *C.* *saccharolyticus* and *C.* *owensensis.*

**Table 6:** PCR primers for *C.* *saccharolyticus* and *C.* *owensensis* differentiation

Species	Primer	Seqeunce
*C.* *owensensis*	Cowen_F1	5′-GGCAAGTGGGAAGAAGATGA-3′
*C.* *owensensis*	Cowen_R1	5′-CTCCGCAAGACTTGAACACA-3′
*C.* *saccharolyticus*	Csacc_F1	5′-TATTATGGGGATTGGGACGA-3′
*C.* *saccharolyticus*	Csacc_R1	5′-CTGGCGCACCAAAGATAAAT-3′

Sequences were obtained through multiple genome alignment using Mauve [9]. qPCR reactions were conducted using DreamTaq DNA polymerase (ThermoFisher, Waltham, MA, USA) and EvaGreen® Dye (Biotium, Fremont, CA) in a BioRad CFX96 Realtime PCR (BioRad, Hercules, CA, USA) machine. The Quantification cycle (*C*_q_) values and melting curve analysis were determined using the CFX Manager™ software 3.1 (Bio-Rad, Hercules, CA, USA). The copy numbers obtained in the software by absolute quantification were in relation to defined standard concentrations (0.02 to 20 ng/µL) obtained from known quantities of genomic DNA obtained from pure cultures. The sum of calculated copy number values was used to determine the relative population of the different species. The following PCR conditions were used: denaturation 95 °C 7 min; 32 cycles of 95 °C 30 s, 54 °C and 56 °C for *C.* *owensensis* and *C* *saccharolyticus*, respectively, for 30 s, 70 °C 20 s; melting curve analysis: 65 °C 30 s hold time, ramp to 95 °C with 0.05 °C/s. Each sample was analyzed in biological duplicates.

### Mathematical modeling

To quantify and evaluate the effect of the sugar concentration, expressed as the osmolarity, on the parental and adapted strains, a kinetic mathematical model was adapted from Ljunggren et al. [21] and run in MATLAB R2017a (Mathworks, USA). The model was set up on a molar basis containing mathematical expressions for microbial growth, substrate consumption, product formation and gas to liquid mass transfer. The model was used with a few alterations to the mass balance equations. The mass balances of the gaseous compounds hydrogen and carbon dioxide are expressed as a change in concentration (mol/L) over time instead of a change in flow over time. This is similar to what has been described in Björkmalm et al. [4] and given as the following equations:

2 $$ \frac{{{\text{dH}}_{{2,{\text{g}}}} }}{{{\text{dt}}}} = \frac{{V_{{{\text{liq}}}} }}{{V_{{{\text{gas}}}} }}*\rho_{{{\text{t}},{\text{H}}_{2} }} + \left( { - {\text{H}}_{{2,{\text{g}}}} \cdot \frac{{q_{{{\text{gas}}}} }}{{V_{{{\text{gas}}}} }} } \right), $$

3 $$ \frac{{{\text{dCO}}_{{2,{\text{g}}}} }}{{{\text{dt}}}} = \frac{{V_{{{\text{liq}}}} }}{{V_{{{\text{gas}}}} }}*\rho_{{{\text{t}},{\text{CO}}_{2} }} + \left( { - {\text{CO}}_{{2,{\text{g}}}} \cdot \frac{{q_{{{\text{gas}}}} }}{{V_{{{\text{gas}}}} }} } \right), $$

where *V*_liq_ and *V*_gas_ are the liquid and the gas volumes (L), respectively, *q*_gas_ is the total gas flow (L/h), H_2,g_ is gaseous hydrogen (mol/L), CO_2_ is gaseous carbon dioxide (mol/L), $\rho_{{{\text{t}},{\text{H}}_{2} }}$ and $\rho_{{{\text{t}},{\text{CO}}_{2} }}$ are the mass transfer rate of hydrogen and carbon dioxide, respectively (mol/L/h).

The osmolarity expression, Eq. 5, is calculated in the same way as Ljunggren et al. [21], except that CO_2,sol_, i.e., the CO_2_ ionic species (bicarbonate and carbonate), is excluded since these were not measured experimentally. This is further motivated by the fact that, according to model calculations in the current study, CO_2,sol_ constituted to less than 2% of the total osmolarity:

4 $$ {\text{OSM}} = {\text{Glu}} + 2 \cdot {\text{Ac}} + 2 \cdot {\text{Lac}} + 0.08, $$

where Glu, Ac and Lac are the concentrations of glucose, acetate and lactate, respectively. 0.08 is the estimated background osmolarity of the medium and it is adjusted slightly in comparison to the benchmark value from Ljunggren et al. [21]. The background osmolarity has not been experimentally measured in this case. The stoichiometric factor 2 implies that for each mole of acid produced, one mole of NaOH is included that was added to maintain the pH.

The inhibition due to osmolarity and dissolved hydrogen concentration is expressed as [21]:

5 $$ I_{{{\text{osm}}}} = 1 - \left( {\frac{{{\text{OSM}}}}{{{\text{OSM}}_{{{\text{crit}}}} }}} \right)^{{n_{\upmu } }} , $$

6 $$ I_{{{\text{H}}_{{2,{\text{aq}}}} }} = 1 - \left( {\frac{{{\text{H}}_{{2,{\text{aq}}}} }}{{{\text{H}}_{{2,{\text{aq}},{\text{crit}}}} }}} \right)^{{n_{{{\text{H}}_{2} }} }} , $$

which are implemented in the growth kinetic equation:

7 $$ \mu = \mu_{\max } \cdot \frac{S}{{S + K_{{\text{s}}} }} \cdot I_{{{\text{osm}}}} \cdot I_{{{\text{H}}_{{2,{\text{aq}}}} }} , $$

where *n*_µ_ and $n_{{{\text{H}}_{2} }}$ are exponential parameters describing the degree of inhibition and OSM_crit_ (mol/L) and H_2,aq,crit_ (mol/L) are the critical osmolarity and critical dissolved hydrogen concentration, respectively. OSM_crit_ is central in this context where a high value of OSM_crit_ indicates a high tolerance for osmolarity. *µ* (h^−1^) is the specific growth rate, *µ*_max_ (h^−1^) is the maximum specific growth rate, *K*_S_ (mol/L) is the affinity constant for glucose and *S* (mol/L) is the concentration of glucose. The mass balance equation for the biomass *X* consists of the rate of glucose consumption *r*_s_ (cmol/L/h), with *Y*_*S*,*X*_ (cmol/mol) as the yield of biomass from glucose, and the cell death rate, *r*_cd_ (h^−1^), which is based on first-order kinetics.

The model was evaluated against different batch experimental data. To fit the model to experimental data, a parameter calibration was conducted using the function *lsqcurvefit* in MATLAB. This function solves the nonlinear curve-fitting problem using the least-square method. The parameters considered to be of greatest importance were *µ*_max_, OSM_crit_, *r*_cd_, $Y_{{S,{\text{H}}_{2} }}$ (H_2_ yield coefficient, mol H_2_/mol glucose), *n*_µ_ and $n_{{{\text{H}}_{2} }}$. The MATLAB function *nlparci* was used to calculate the 95% confidence interval for the calibrated parameters to assess their uncertainties.

To assess the accuracy of the model in relation to the experimental data, *R*^2^ values and curve slope values were calculated. This was done by plotting the simulated values against the experimental values followed by a linear regression which gave the *R*^2^ value as well as the linear equation *y* = *k*·*x*, where *k* is the curve slope value.

When calibrating the parameters in the model to get a good fit to the experimental data, an initial start value of the parameter needs to be guesstimated. These values are of great importance for the end result as a poorly chosen initial value could result in a local minimum in the parameter estimation procedure, leading to a bad fit of the model to the experimental data and a faulty estimated parameter. To counteract this, the start values were initially chosen in proximity to the benchmark values from Ljunggren et al. [21]. When these values did not give the right fit to the experimental data, several new initial start values were tested as input in the *lsqcurvefit* function in MATLAB.

The biomass yield coefficient *Y*_*S*,*X*_ was calculated using the experimental data, but altered in the 80 g/L model to fit the experimental data. The yields for hydrogen, acetate, lactate and carbon dioxide used in the model, $Y_{{S,{\text{H}}_{2} }} ,$ *Y*_*S*,Ac_, *Y*_*S*,Lac_ and $Y_{{S,{\text{CO}}_{2} }}$, were based on stoichiometry according to:

8 $$ {\text{C}}_{6} {\text{H}}_{12} {\text{O}}_{6} + 2{\text{H}}_{2} {\text{O}} \to 2{\text{C}}_{2} {\text{H}}_{4} {\text{O}}_{2} + 2{\text{CO}}_{2} + 4{\text{H}}_{2} , $$

9 $$ {\text{C}}_{6} {\text{H}}_{12} {\text{O}}_{6} \to 2{\text{C}}_{3} {\text{H}}_{6} {\text{O}}_{3} . $$

## Data Availability

All data generated or analyzed during this study are included in this article. If additional information is needed, please contact the corresponding author.

## References

[1] Azimian L, Bassi A, Mercer SM (2019). Investigation of growth kinetics of *Debaryomyces hansenii* (LAF-3 10 U) in petroleum refinery desalter effluent. Can J Chem Eng.

[2] Azwar MY, Hussain MA, Abdul-Wahab AK (2014). Development of biohydrogen production by photobiological, fermentation and electrochemical processes: a review. Renew Sustain Energy Rev.

[3] Basen M, Rhaesa AM, Kataeva I, Prybol CJ, Scott IM, Poole FL, Adams MWW (2014). Degradation of high loads of crystalline cellulose and of unpretreated plant biomass by the thermophilic bacterium Caldicellulosiruptor bescii. Bioresource Technol..

[4] Björkmalm J, Byrne E, van Niel EWJ, Willquist K (2018). A non-linear model of hydrogen production by *Caldicellulosiruptor saccharolyticus* for diauxic-like consumption of lignocellulosic sugar mixtures. Biotechnol Biofuels.

[5] Byrne E, Kovacs K, van Niel EWJ, Willquist K, Svensson S-E, Kreuger E (2018). Reduced use of phosphorus and water in sequential dark fermentation and anaerobic digestion of wheat straw and the application of ensiled steam-pretreated lucerne as a macronutrient provider in anaerobic digestion. Biotechnol Biofuels.

[6] Cheng K, Zheng W, Chen H, Zhang Y-HPJ (2019). Upgrade of wood sugar D-xylose to a value-added nutraceutical by in vitro metabolic engineering. Metab Eng..

[7] Ciranna A, Ferrari R, Santala V, Karp M (2014). Inhibitory effects of substrate and soluble end products on biohydrogen production of the alkalithermophile Caloramator celer: kinetic, metabolic and transcription analyses. Int J Hydrog Energy.

[8] Claassen PAM, van Lier JB, Lopez Contreras AM, van Niel EWJ, Sijtsma L, Stams AJM, de Vries SS, Weusthuis RA (1999). Utilisation of biomass for the supply of energy carriers. Appl Microbiol Biotechnol.

[9] Darling ACE, Mau B, Blattner FR, Perna NT (2004). Mauve: multiple alignment of conserved genomic sequence with rearrangements. Genome Res.

[10] de Vrije T, Bakker RR, Budde MA, Lai MH, Mars AE, Claassen PA (2009). Efficient hydrogen production from the lignocellulosic energy crop *Miscanthus* by the extreme thermophilic bacteria *Caldicellulosiruptor saccharolyticus* and *Thermotoga neapolitana*. Biotechnol Biofuels.

[11] de Vrije T, Mars AE, Budde MAW, Lai MH, Dijkema C, de Waard P, Claassen PAM (2007). Glycolytic pathway and hydrogen yield studies of the extreme thermophile *Caldicellulosiruptor saccharolyticus*. Appl Microbiol Biotechnol.

[12] Dötsch A, Severin J, Alt W, Galinski EA, Kreft J-U (2008). A mathematical model for growth and osmoregulation in halophilic bacteria. Microbiology.

[13] Dragosits M, Mattanovich D (2013). Adaptive laboratory evolution—principles and applications for biotechnology. Microb Cell Fact.

[14] European Parliament and Council. Directive (EU) 2015/1513 of the European parliament and of the council of 9 September 2015 amending Directive 98/70/EC relating to the quality of petrol and diesel fuels and amending Directive 2009/28/EC on the promotion of the use of energy from renewable sources. Off J Eur Union. 2015.

[15] Farkas J, Chung D, Cha M, Copeland J, Grayeski P, Westpheling J (2013). Improved growth media and culture techniques for genetic analysis and assessment of biomass utilization by Caldicellulosiruptor bescii. J Industr Microbiol Biotechnol..

[16] Foglia D, Ljunggren M, Wukovits W, Friedl A, Zacchi G, Urbaniec K, Markowski M (2010). Integration studies on a two-stage fermentation process for the production of biohydrogen. J Clean Prod.

[17] Gonçalves LG, Borges N, Serra F, Fernandes PL, Dopazo H, Santos H (2012). Evolution of the biosynthesis of di-myo-inositol phosphate, a marker of adaptation to hot marine environments. Environ Microbiol.

[18] Kempf B, Bremer E (1998). Uptake and synthesis of compatible solutes as microbial stress responses to high-osmolality environments. Arch Microbiol.

[19] Kim J-H, Block DE, Mills DA (2010). Simultaneous consumption of pentose and hexose sugars: an optimal microbial phenotype for efficient fermentation of lignocellulosic biomass. Appl Microbiol Biotechnol.

[20] Krahe M, Antranikian G, Märkl H (1996). Fermentation of extremophilic microorganisms. FEMS Microbiol Rev..

[21] Ljunggren M, Willquist K, Zacchi G, van Niel EW (2011). A kinetic model for quantitative evaluation of the effect of hydrogen and osmolarity on hydrogen production by *Caldicellulosiruptor saccharolyticus*. Biotechnol Biofuels.

[22] Ljunggren M, Zacchi G (2010). Techno-economic analysis of a two-step biological process producing hydrogen and methane. Bioresour Technol.

[23] Lv Z, Zhou J, Zhang Y, Zhou X, Xu N, Xin F, Ma J, Jiang M, Dong W (2020). Techniques for enhancing the tolerance of industrial microbes to abiotic stresses: a review. Biotechnol Appl Biochem.

[24] Martins LO, Santos H (1995). Accumulation of mannosylglycerate and di-myo-inositol-phosphate by *Pyrococcus furiosus* in response to salinity and temperature. Appl Environ Microbiol.

[25] Nunes OC, Manaia CM, Da Costa MS, Santos H (1995). Compatible solutes in the thermophilic bacteria *Rhodothermus marinu*s and “*Thermus thermophilus*”. Appl Environ Microbiol.

[26] Panagiotopoulos IA, Bakker RR, de Vrije T, Koukios EG, Claassen PAM (2010). Pretreatment of sweet sorghum bagasse for hydrogen production by *Caldicellulosiruptor saccharolyticus*. Int J Hydrog Energy.

[27] Pawar SS. *Caldicellulosiruptor saccharolyticus*: an ideal hydrogen producer? In: Faculty of Engineering, LTH, vol. Ph.D. Thesis, Lund University, Lund. 2014.

[28] Pawar SS, Nkemka VN, Zeidan AA, Murto M, van Niel EWJ (2013). Biohydrogen production from wheat straw hydrolysate using *Caldicellulosiruptor saccharolyticus* followed by biogas production in a two-step uncoupled process. Int J Hydrog Energy.

[29] Pawar SS, van Niel EWJ (2014). Evaluation of assimilatory sulphur metabolism in *Caldicellulosiruptor saccharolyticus*. Bioresour Technol.

[30] Pawar SS, Vongkumpeang T, Grey C, van Niel EW (2015). Biofilm formation by designed co-cultures of *Caldicellulosiruptor* species as a means to improve hydrogen productivity. Biotechnol Biofuels.

[31] Peabody G, Winkler J, Fountain W, Castro DA, Leiva-Aravena E, Kao KC (2016). Benefits of a recombination-proficient *Escherichia coli* system for adaptive laboratory evolution. Appl Environ Microbiol.

[32] Peintner C, Zeidan AA, Schnitzhofer W (2010). Bioreactor systems for thermophilic fermentative hydrogen production: evaluation and comparison of appropriate systems. J Clean Prod.

[33] Rainey FA, Donnison AM, Janssen PH, Saul D, Rodrigo A, Bergquist PL, Daniel RM, Stackebrandt E, Morgan HW (1994). Description of *Caldicellulosiruptor saccharolyticus* gen. nov., sp. nov.: an obligately anaerobic, extremely thermophilic, cellulolytic bacterium. FEMS Microbiol Lett.

[34] Rodrigues MV, Borges N, Almeida CP, Lamosa P, Santos H (2009). A unique β-1,2-mannosyltransferase of *Thermotoga maritima* that uses di-myo-inositol phosphate as the mannosyl acceptor. J Bacteriol.

[35] Sandberg TE, Salazar MJ, Weng LL, Palsson BO, Feist AM (2019). The emergence of adaptive laboratory evolution as an efficient tool for biological discovery and industrial biotechnology. Metab Eng..

[36] Sander KB, Chung D, Klingeman DM, Giannone RJ, Rodriguez M, Whitham J, Hettich RL, Davison BH, Westpheling J, Brown SD (2020). Gene targets for engineering osmotolerance in *Caldicellulosiruptor bescii*. Biotechnol Biofuels.

[37] Sanderson K (2011). Lignocellulose: a chewy problem. Nature.

[38] Schleifer KH (2009). Phylum XIII. Firmicutes Gibbons and Murray 1978, 5 (Firmicutes [sic] Gibbons and Murray 1978, 5). Bergey’s manual® of systematic bacteriology.

[39] Sims R, Taylor M, Saddler J, Mabee W (2008). From 1st-to 2nd-generation biofuel technologies.

[40] Sivakumar A, Srinivasaraghavan T, Swaminathan T, Baradarajan A (1994). Extended monod kinetics for substrate inhibited systems. Bioprocess Eng.

[41] Tomás A, Karakashev D, Angelidaki I (2011). Effect of xylose and nutrients concentration on ethanol production by a newly isolated extreme thermophilic bacterium. Water Sci Technol..

[42] Tomás AF, Karakashev D, Angelidaki I (2013). *Thermoanaerobacter pentosaceus* sp. nov., an anaerobic, extremely thermophilic, high ethanol-yielding bacterium isolated from household waste. Int J Syst Evolut Microbiol.

[43] van Niel EWJ, Claassen PAM, Stams AJM (2003). Substrate and product inhibition of hydrogen production by the extreme thermophile, *Caldicellulosiruptor saccharolyticus*. Biotechnol Bioeng.

[44] VanFossen AL, Verhaart MRA, Kengen SMW, Kelly RM (2009). Carbohydrate utilization patterns for the extremely thermophilic bacterium *Caldicellulosiruptor saccharolyticus* reveal broad growth substrate preferences. Appl Environ Microbiol.

[45] Willquist K, Claassen PAM, van Niel EWJ (2009). Evaluation of the influence of CO_2_ on hydrogen production by *Caldicellulosiruptor saccharolyticus*. Int J Hydrog Energy.

[46] Zeidan AA, van Niel EWJ (2010). A quantitative analysis of hydrogen production efficiency of the extreme thermophile *Caldicellulosiruptor owensensis* OLT. Int J Hydrog Energy.

